# Growth, death, and resource competition in sessile organisms

**DOI:** 10.1073/pnas.2020424118

**Published:** 2021-04-09

**Authors:** Edward D. Lee, Christopher P. Kempes, Geoffrey B. West

**Affiliations:** ^a^Santa Fe Institute, Santa Fe, NM 87501

**Keywords:** ecology, population dynamics, spatial patterning, metabolic scaling, sessile

## Abstract

Population-level scaling in ecological systems arises from individual growth and death with competitive constraints. We build on a minimal dynamical model of metabolic growth where the tension between individual growth and mortality determines population size distribution. We then separately include resource competition based on shared capture area. By varying rates of growth, death, and competitive attrition, we connect regular and random spatial patterns across sessile organisms from forests to ants, termites, and fairy circles. Then, we consider transient temporal dynamics in the context of asymmetric competition, such as canopy shading or large colony dominance, whose effects primarily weaken the smaller of two competitors. When such competition couples slow timescales of growth to fast competitive death, it generates population shocks and demographic oscillations similar to those observed in forest data. Our minimal quantitative theory unifies spatiotemporal patterns across sessile organisms through local competition mediated by the laws of metabolic growth, which in turn, are the result of long-term evolutionary dynamics.

## Significance

Although termite mounds stand out as an example of remarkably regular patterns emerging over long times from local interactions, ecological spatial patterns range from regular to random, and temporal patterns range from transient to stable. We propose a minimal quantitative framework to unify this variety by accounting for how quickly sessile organisms grow and die mediated by competition for fluctuating resources. Building on metabolic scaling theory for forests, we reproduce a wide range of spatial patterns and predict transient features such as population shock waves that align with observations. By connecting diverse ecological dynamics, our work will help apply lessons from model systems more broadly (e.g., by leveraging remote mapping to infer local ecological conditions).

**Fig. 1.**
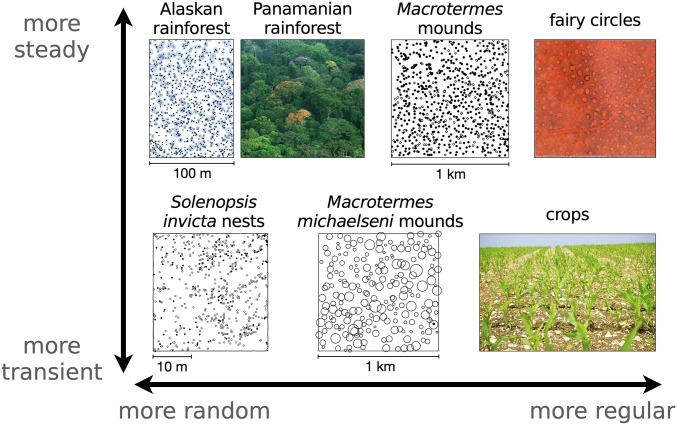
Regular to random spatial distributions and transient to slow temporal evolution in sessile organisms. (*Upper*) Trees in Alaskan rainforest (circles indicate basal stem diameter of >2.5 cm increased by a factor of five) (5), view of the Panamian rainforest canopy, semiregularly packed termite mounds reprinted from ref. 3 (empty circles are inactive mounds), and hexagonally packed fairy circles reprinted from ref. 1. (*Lower*) Newly built ant nests (4), termite mounds with size shown by circles (2), and perennial agricultural crops. Dynamics range from transience dominated, in the case of crops razed at the end of the season or newly built ant nests that die within days as indicated by open circles, to long-lasting structures such as fairy circles, which can live individually for decades or forests at demographic equilibrium lasting millennia. Scale is unavailable for fairy circles, but they range from 2 to 12 m in diameter, meaning that the shown plot covers some hundreds of meters on a side (1). Panamanian rainforest image credit: Christian Ziegler (photographer). Termite mounds image reprinted from ref. 3, which is licensed under CC BY 4.0. Fairy circles image reprinted from ref. 1, which is licensed under CC BY 4.0. *Solenopsis invicta* nests image reprinted by permission of ref. 4: Springer Nature, *Oecologia*, copyright 1995. *M. michaelseni* mounds image reprinted by permission of ref. 2: Springer Nature, *Insectes Sociaux*, copyright 2010. Crops image credit: Pxhere.

Ecological niches display a wide variety of spatial and temporal patterns ranging from random to regular and from transient to long lived. In Fig. 1, we show a small sample from such diversity, including fairy circles in semiarid environments (1), regular and random tiling of termite mounds (2, 3), and more randomly spaced ant nests and trees (4, 5). This variation is not limited to between taxa but also varies between different plots in the same region. These systems also operate on different timescales, where fairy circles have estimated lifetimes of around half of a century compared with days or weeks for nascent ant nests and centuries for trees in unperturbed forests. In the extreme, transient growth is maximized for agricultural crops, which are then razed at maturity before demographic stability (6, 7). Overall, fast and slow dynamics of sessile organisms are characterized by a range of spatial distributions, from the random to the regular, that reflect underlying forces of growth, death, and competition.

The mechanisms underlying such pattern formation have been a source of robust debate, especially in the context of vegetation (8, 9). Following Turing’s seminal work on scale-dependent feedback, namely local activation and long-range inhibition, similar principles of pattern formation with local density dependence have been considered (10–13), touching on the more general question of how multiple scales of time and space emerge (14–16). More recent work has connected these principles with mechanisms of biological interaction and environmental feedback (17–19). For spatial patterning, approaches to mechanism range from using perturbations like cascades of tree death to explore self-organized criticality in forests (20–22) to applying Turing-like activation-inhibition concepts to scale-dependent plant processes (15, 16), which could be modulated by environmental conditions (23), to considering how ecosystem engineers modify the local environment to generate bare and densely vegetated patches (18, 24). Demographic theories, in contrast, focus on variables that aggregate across species and space such as age and size (25–27) and build on allometric dependence of growth, mortality, and resource acquisition (28–36). In an alternative set of approaches, mechanism-free maximum entropy principles can capture demographic patterns by fixing a few population “state variables” to predict measured properties (37). Across these examples, forests are particularly well-studied empirically across diverse species, sizes, and environments (38, 39) and grounded on predicted theoretical regularities in space and demography such as in the context of metabolic scaling (40–43) and mechanical or hydraulic limits (44–47).

Here, we build on previous work on forest growth and structure to consider sessile organisms more broadly in the context of both spatial structure and demographic dynamics. We propose a minimal dynamical model that integrates timescales of individual growth and mortality with competitive attrition on a background of fluctuating resources. With the model, we study the emergence and erasure of spatiotemporal order in ecological systems. We show how competition alone is insufficient to generate strong spatial regularity and that growth and death must flatline for the largest organisms to stabilize spatial order. Since most ecological systems are out of equilibrium, we extend our model to consider transient phenomena and predict population shock waves from competitive interactions when there is metabolic growth. This minimal framework serves to unify at a conceptual level the role of various timescales for pattern formation in distinct ecological settings.

As the starting point, we consider how metabolism determines individual growth and death. Metabolic scaling theory describes the origins of scaling laws in organism growth across a large range of body sizes derived from energetic constraints (40, 48, 49). Given constraints on average resource consumption per unit area, individual growth follows power law, allometric scaling relations connecting accumulation of biomass m, or the organism’s physical dimensions such as the stem radius r with age. In the context of forests where individuals are fixed in location, metabolic scaling can be connected with population-level statistics such as spatial density, biomass production, and stand energetics determined by the balance of individual growth and mortality (34, 43). Such predictions have been verified for individual organisms (41, 50) and have highlighted ecosystem-level regularities such as total population density and predator–prey relations (51, 52). The presence of universal patterns suggests that unifying principles act across systems (53, 54) such as from energetic constraints (34, 43, 55, 56). Such observations form the basis of using power law, allometric relationships to describe the rates of processes, but this is not essential. Any number of assumptions or mathematical relationships could replace these in our overall framework.

One surprising prediction of metabolic scaling theory is that it is not necessary to explicitly include local competitive interactions to explain steady-state population distributions (33, 34), even if local competition is one of the major factors that drives long-term evolutionary dynamics to optimize fundamental energetic constraints (57). However, competition coupled with other timescales can introduce complex dynamics (58, 59) such as in response to exogenous perturbations (60, 61), which goes beyond steady-state assumptions. Other than mechanistic additions to metabolic scaling theory (44), competition, perturbation, and other dynamics present potential explanations for significant and sometimes substantial deviations from predictions (39, 42). Here, we present a minimal model to account for these missing factors.

We start with allometric scaling theory of forest growth in section 1 and connect deviations from metabolic scaling theory to organism density, resource variability, and competitive interactions in section 2. We explore the implications of competition through space in section 3 and time in section 4, concluding with section 5. Although we explicitly develop our framework using the language of forests, referring, for instance, to individuals as trees and dimensions as stem radii, our formulation is straightforwardly generalizable to other sessile organisms (62). As an example, we extend our model beyond allometric assumptions to consider the emergence of spatial order (*SI Appendix*, section D).

## 1. A Size-Class Model for Population Growth

The fractal structure of a forest exists both at the level of the physical branching of individual trees and at the level of self-similar packing of differently sized individuals. This fractal structure reflects energetic constraints that have shaped the long-term evolutionary dynamics of forest life (33, 50, 63). Connecting energy expenditure with the physical limits of how vasculature distributes energy leads to an allometric scaling theory of growth. For the rate of basal stem radius growth, $\dot{r}$, we have (40, 48)

$$\dot{r}\left(r\right)\approx \frac{3}{8}c_{m}^{1-b}ār^{b}.$$ [1]

For sufficiently long times, $r∼t^{1/\left(1-b\right)}$ for time t. Eq. **1** expresses the general principle of biomass production in terms of a constant determined from biological energetics ā, how radius scales with tree mass m, $r=c_{m}m^{3/8}$, and a growth exponent b=1/3. Other sessile organisms fill available space determined by analogous mechanisms of growth, death, and competition, suggesting that metabolic principles reflecting vascular or other constraints (with differing allometric exponents) bridge well-studied forests and sessile organisms more generally (8).

Building on the metabolic picture of growing individuals, we consider size classes labeled by radial dimension $r_{k}$ with index k of population number $n_{k}\left(t\right)$, which is a function of time t. Using forests as our example, these size classes group together trees of various species, roles, and microenvironments, and so we describe properties averaged over such variety. The smallest size class k=0 is filled with saplings of stem radius $r_{0}$ that have grown from seedlings with rate $g_{0}$ (42). As new saplings appear in the system, older ones grow into the next class k=1, reflected in the rate of change of stem radius ${\dot{r}}_{k}$, where the discrete classes encompass stems of radius within the interval $\left[r_{k},r_{k}+\Delta r\right)$. Furthermore, trees die with a size-dependent inherent mortality rate $\mu_{k}$, which we consider independent of competition-based mortality. Accounting for these individual properties of metabolic growth and death, we obtain a dynamical equation for change in population number per unit time for saplings:

$$\partial_{t}n_{0}\left(t\right)=g_{0}-n_{0}\left(t\right)\left[{\dot{r}}_{0}/\Delta r+\mu_{0}\right].$$ [2]

For larger trees, the change in the population is determined by the rate at which smaller plants in size class k−1 grow into the size class k,

$$\partial_{t}n_{k}=n_{k-1}{\dot{r}}_{k-1}/\Delta r-n_{k}\left[{\dot{r}}_{k}/\Delta r+\mu_{k}\right],$$ [3]

describing a sequence of ever larger tree sizes that are populated by an incoming flux of younger and smaller trees and depopulated as trees grow to a larger size or die. Thus, Eqs. **2** and **3**, without specifying the particular functional forms for growth ${\dot{r}}_{k}$ and mortality $\mu_{k}$, describe the simplest possible form for independent tree growth without reference to either environment or local competitors.

Although population is typically binned into discrete size classes in both observation and theory, tree growth is in reality a function of continuous radius r. Relating the index k to radius r such that $r_{k}\equiv r_{0}+k\;\Delta r$, we obtain

$$\partial_{t}n\left(r,t\right)=-\partial_{r}\left[n\left(r,t\right)\dot{r}\left(r\right)\right]-n\left(r,t\right)\mu \left(r\right)$$ [4]

with sapling boundary condition

$$\partial_{t}n\left(r_{0},t\right)=g_{0}-n\left(r_{0},t\right)\left[\dot{r}\left(r_{0}\right)/\Delta r+\mu \left(r_{0}\right)\right],$$ [5]

also known as demographic equilibrium theory when referring to the steady state (32) (*SI Appendix*, section A). These equations determine the continuum formulation of the size-class model, including only growth and natural mortality as a starting hypothesis.

Taking predictions from allometric scaling theory that relate mass growth function dm/dt with tree radius in Eq. **1**, we obtain a functional form for mortality (34, 39, 64). With Eq. **1** and $n\left(r\right)\propto r^{-\alpha }$ and at stationarity $\partial_{t}n\left(r,t\right)=0$,

$$\begin{array}{cc} \mu \left(r\right) & =Ār^{b-1},\\ Ā & =\frac{3}{8}āc_{m}^{1-b}\left[\alpha -b\right]. \end{array}$$ [6]

Thus, stationarity directly fixes the form of metabolic mortality in the simple size-class model from Eqs. **4** and **5** in a way that determines the population number exponent:

$$\alpha =b+\frac{8Ā}{3āc_{m}^{1-b}}.$$ [7]

The population number exponent in Eq. **7** indicates the role of metabolic growth in the first term and the relative timescales of growth and death in the second. When growth dominates, we would recover α≈b=1/3, and population number is determined solely by the growth curve, whereas when mortality overtakes individuals quickly, α→∞, no trees survive beyond birth. When metabolic growth is determined by a power law, the simple size-class model fixes the forms of scaling in mortality and population as a combination of both the exponent driving growth but also the relative timescales at which mortality and growth act (32).

From this minimal model of tree growth under the scaling assumptions of individual tree allometry, we obtain a wide range of possible steady states encompassing both predictions consistent with metabolic scaling theory as well as virtually any other population number scaling. This reflects the fact that space filling in forests, when α=2, does not depend separately on typical growth and mortality rates but is determined by the ratio of the scaling coefficients, which may be fixed by energetic constraints. Since these features only determine the exponent, deviations from space filling at steady state (such as for size distributions observed in large trees [figure 1 in ref. 34]) could arise from processes such as competitive interactions, which are not included in a model only accounting for metabolic scaling.

## 2. Competition for Fluctuating Resources

Resource collection in sessile organisms is fundamentally connected to local area (62). Examples include 1) local foraging by ants and termites (18); 2) diffusive depletion zones that regulate biofilm and microbial mat growth and spatial patterning (65–67); and 3) nutrients, water, and sunlight collection in trees through overlapping root or canopy volumes (49, 68), where competition is largely determined by area overlap between neighbors (19). Overlapping canopies in particular reduce light available to shorter trees but not to taller ones (36), an example of asymmetric competitive interaction that we discuss later. As a general formulation of the consequences of symmetric competitive interactions (4), we consider how resource availability is modulated by overlap and environmental fluctuations relative to basal metabolic need.

All organisms have some basal resource budget $Q_{0}$ above which growth is feasible. For local resource capture, we expect the budget to scale with physical dimension to some exponent $\eta_{1}$ and constant parameter $\beta_{1}$, or $Q_{0}\left(r\right)=\beta_{1}r^{\eta_{1}}$, inspired by observations for trees (49).* If the total amount of resource per unit area is a time-fluctuating quantity ρ(t), then the amount of resource that tree i could potentially obtain from a resource area, $a_{i}\equiv a\left(r_{i}\right)\propto r^{2\alpha_{r}}$, is $\rho \left(t\right)a_{i}$. Beyond periodic diurnal patterns, long-time averaged resource distribution fluctuates randomly about the mean $\bar{\rho }$, captured by division with a random variable ξ representing scarcity, $\rho \left(t\right)=\bar{\rho }/\xi \left(t\right)$. Noting that in some cases—such as durations of low precipitation (69) (*SI Appendix*, Fig. S2)—resource fluctuations can be modeled accurately with power law tails, we consider a scale-free distribution of fluctuations $h\left(\xi \right)=\xi_{0}^{1-\nu }\xi^{-\nu }$, where $\xi_{0}$ ensures that $\bar{\xi }=1$.^†^ The exponent ν primarily summarizes whether extreme events dominate the distribution 1<ν<2 or fluctuations are tightly limited ν>2. Although time-averaged resource availability may determine maximum tree size (49), it is the fluctuations below basal metabolic requirements that induce mortality.

Putting these together, incoming resource rate depends on the amount of sharing that tree i with resource area $a_{i}$ does with neighbor j given the overlap in area, $\Delta_{i}a_{j}$:

$$\Delta Q\left(t\right)=\varepsilon \rho \left(t\right)a_{i}\left[1-f\underset{\left\langle ij\right\rangle }{\sum }\Delta_{i}a_{j}\right]-Q_{0}\left(r_{i}\right).$$ [8]

Eq. **8** indicates a resource extraction efficiency ε, a sum over the neighbors $<ij>$ indexed j of tree i, and a constant fraction f∈[0,1] of resources siphoned off given overlap with each competing neighbor. When f=1/2, competitors equally split available resources, a zero-sum game; however, for f>1/2, competition reduces resource availability overall as if individuals pay a cost for competing, and for f<1/2, resources are reusable or the relationship is symbiotic. When ΔQ<0, such as with large overlap or high scarcity, mortality from resource stress occurs with rate s such that trees are sensitive to resource deprivation when s≫1 and relatively robust to such fluctuations when s≪1. Thus, Eq. **2** captures the balance of basal metabolic needs with resource competition that strengthens with overlapping area.

Averaging over many spatial arrangements over a long period of time, we consider the mean-field effect from such competition (*SI Appendix*, section C has details):

$$\Delta \bar{Q}\left(t\right)=\varepsilon \rho \left(t\right)a_{i}\left[1-f\right]-Q_{0}\left(r_{i}\right).$$ [9]

Thus, resource competition with neighbors saps fraction f from total incoming resource flux at any given time $\varepsilon \rho \left(t\right)a_{i}$. The approximation in Eq. **9** is an accurate description when considering many trees over a large area that just fill the available space and interact weakly, but it assumes that interactions are stronger than in two dimensions. Then, competitive attrition rate matters when incoming resources are insufficient to cover basal metabolic rate. With probability $p\left(\xi >\xi_{basal}\right)=\int_{\xi_{basal}}^{\infty }h\left(\xi \right)\;d\xi$, such insufficiency occurs, where $\Delta \bar{Q}=0$ defines a minimum sustainable level of scarcity $\xi_{basal}$. The resulting probability of fatal fluctuations is

$$\begin{array}{cc} p\left(\xi >\xi_{basal}\right) & =Br^{\kappa },\\ B=r_{\text{max}}^{-\kappa }, & \;\kappa =\left(\nu -1\right)\left(\eta_{1}-2\alpha_{r}\right). \end{array}$$ [10]

The probability is normalized by constant B set by recognizing that there is only a single largest tree with radius $r_{\max}$ by definition, which then relates the phenomenological parameters ε and f. Note that we expect κ>0. When resource area grows slower than metabolic need, as is the case for $\eta_{1}>2\alpha_{r}$, larger trees have less margin for low resources because they sit close to the boundary of basal metabolic need. Yet, if it were possible (although unrealistic) for resource area to grow faster, $\eta_{1}<2\alpha_{r}$ and κ<0, then growth is unconstrained, and larger trees have more buffer to withstand environmental fluctuations.^‡^Furthermore, the form of exponent κ shows that growth in metabolic cost is mediated by the fluctuations in resource availability described by exponent ν such that when ν>2, its distribution is narrow, and we expect there to be sharp difference in the impact of competition for large trees below and above a cutoff. For ν→1, all trees, small or large, will pay substantial costs for competition.

Combining metabolic growth and mortality from Eqs. **4** and **5** and competition from Eq. **10**, we obtain the generalized size-class model

$$\begin{array}{cc} \partial_{t}n\left(r,t\right) & =-\partial_{r}\left[n\left(r,t\right)\;\dot{r}\left(r\right)\right]-n\left(r,t\right)\left[\mu \left(r\right)+Bs\;r^{\kappa }\right]. \end{array}$$ [11]

By solving Eq. **11** for steady state, we find

$$n\left(r\right)={ñ}_{0}{\left(\frac{r}{r_{0}}\right)}^{-\alpha }\exp\left(-\frac{F}{\kappa +1-b}r^{\kappa +1-b}\right),$$ [12]

with normalization constant ${ñ}_{0}$ and $F\equiv 8Bsc_{m}^{b-1}/3ā$. This shows that an exponentially decaying tail truncates the simple scaling form, imposing a cutoff on a scale of ${\left[F/\left(\kappa +1-b\right)\right]}^{-1/\left(\kappa +1-b\right)}$. Eq. **11** is the general formulation incorporating both metabolic scaling theory and the impact of area-mediated resource use from pairwise competitive interactions, yielding the product of a scaling law with a decaying tail that wiggles as resource fluctuations are varied.

**Fig. 2.**
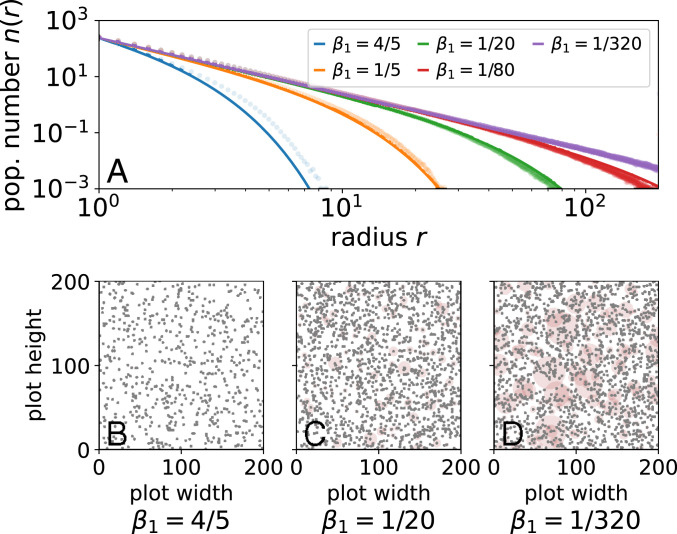
(*A*) Population number n(r) with varying strength of area competition (α=2, ν=5/2, L=200, averaged over time, and K=15 random forests). Mean-field approximation (solid lines) mirrors the shape of the 2D forest simulation (circles) for varying basal metabolic coefficient $\beta_{1}$. (*B–D*) Simulated forest plots; automaton model details are in *SI Appendix*, section F. Brown circles represent root competition area centered about gray dots.

We show numerical simulations of an explicit two-dimensional (2D) simulation of trees in a large plot in Fig. 2*A* in comparison with the mean-field approximation from Eq. **12**. The mean-field approximation does not exactly capture the tail of the distribution, but it does surprisingly well. Furthermore, it captures the qualitative intuition that for large trees, r≫1, metabolic constraints dominate, introducing an upper cutoff on the maximum possible tree size in the system whose radius varies with the growth coefficient. The suddenness of this cutoff is controlled by the fluctuation exponent ν such that we find strong curvature away from purely scale-free metabolic scaling in the stationary distribution for smaller ν.

Importantly, the mean-field argument clearly links resource area with resource fluctuations. This means that deviations from metabolic scaling theory may result from different combinations of resource-area growth and fluctuations in a way that make effects hard to disentangle (31). Beyond the particular form of competitive interactions we consider, this framework is naturally extensible by, for example, modifying resource-sharing fraction f to reflect cooperative or noncooperative interactions or an allometric dependency. Such modifications do not change underlying metabolic scaling but do change the probability of fatal fluctuations, $p\left(\xi >\xi_{basal}\right)$. Thus, the derived scaling form summarizes a variety of competitive effects in the exponent κ, suggesting how physical scaling might lead to universal, or similar, demographic scaling across different biological systems.

## 3. From Random to Regular Spatial Patterns

Different organisms, and even the same organism in another environment, may show systematic variation in spacing (70). Such variation reflects individual growth dynamics and the strength of resource area-based competition due to the local properties of competitor species or the environment (7, 12, 71). Returning to Fig. 1, we again point out randomness in spatial surveys of an Alaskan rainforest along with *Macrotermes michaelseni* mounds and ant nests. In contrast, the spacing between *Macrotermes falciger* mounds is more regular. Other than intertaxonomic variation, there is also evidence of random and systematic variation between different plots in nearby regions.^§^ Thus, natural spatial distributions of sessile organisms may be attributable to the assorted effects of individual allometries and local competition in our model.

**Fig. 3.**
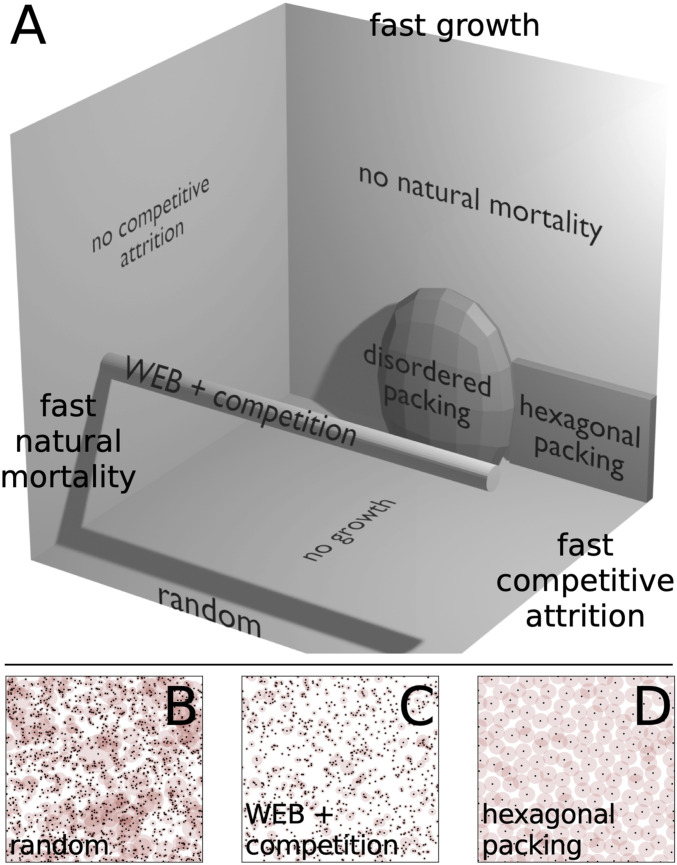
(*A*) Schematic 3D realm of models defined on rates of growth, death, and competitive attrition (shadows are generated by a single-point light source at the upper right). WEB theory of allometric forest growth corresponds to fixing population number exponent α=2 while varying competitive attrition rate (cylinder). Regular hexagonal packing only emerges in a tight limit where growth and death rates approach zero and competitive death rate is high. (*B–D*) By varying timescales, we obtain spatial patterns qualitatively similar to examples in Fig. 1.

We survey such variety in Fig. 3 along a schematic region of spatial patterns generated by our model. We vary the rates of growth, death, and competition in Eq. **11**. The planes jutting out from the back corner in Fig. 3*A* all correspond to theories where one of the terms is negligible. When competitive attrition is negligible, or s→0, population scaling is pinned to the plane where there is a perfect scaling law determined by mortality and growth. For example, the idealized West, Enquist, and Brown (WEB) model for forest distributions contains only growth and death and corresponds to the point where α=2 (33, 34). The other limits of no natural mortality or no growth lead to qualitatively different configurations that may mimic other spatial patterns found across sessile organisms. In this sense, this realm of models is a three-dimensional (3D) slice of a much higher-dimensional set of models with different exponents, as opposed to rates, yet it is sufficient to capture qualitative variety in ecological spatial patterning.

In the limit of weak interaction, the spatial distribution of individuals is random. Then, the probability of not encountering any neighbors within a distance $r_{\text{min}}$ is given by the Poisson distribution with average $\sigma \pi r_{\text{min}}^{2}$, with organism density σ. However, for finite plots like the ones we consider in Fig. 3 and forest plot surveys, it is essential to account for corrections from points sitting near the boundaries. The typical number of points close to the boundaries for a unit square is η=2σ, and these only have half of the typical number of neighbors. As a result, the probability of the nearest neighbor being at distance greater than $r_{\text{min}}$ is the mixture

$$q\left(r_{\text{min}}\right)=\left(1-\eta \right)\exp\left(-\sigma \pi r_{\text{min}}^{2}\right)+\eta \exp\left(-\sigma \pi r_{\text{min}}^{2}/2\right).$$ [13]

Competitive interactions manifest as deviations from the prediction of Eq. **13**. As a measure of difference between the random distribution $q\left(r_{\text{min}}\right)$ and observation $p\left(r_{\text{min}}\right)$, we rely on a principled quantitative measure, the Kullback–Leibler divergence (72):

$$D_{\text{KL}}\left[p||q\right]=\int_{r_{0}}^{\infty }p\left(r_{\text{min}}\right)\log_{2}\left(\frac{p\left(r_{\text{min}}\right)}{q\left(r_{\text{min}}\right)}\right)\;\text{d}r_{\text{min}}.$$ [14]

Calculation of Eq. **14** requires determining a bin size for integration, as is discussed further in *SI Appendix*, section E. Eq. **14** represents a holistic way of measuring the strength of competitive interactions using nearest-neighbor distances in contrast with mean measures like overdispersion that do not account for the shape of the distribution (71).

**Fig. 4.**
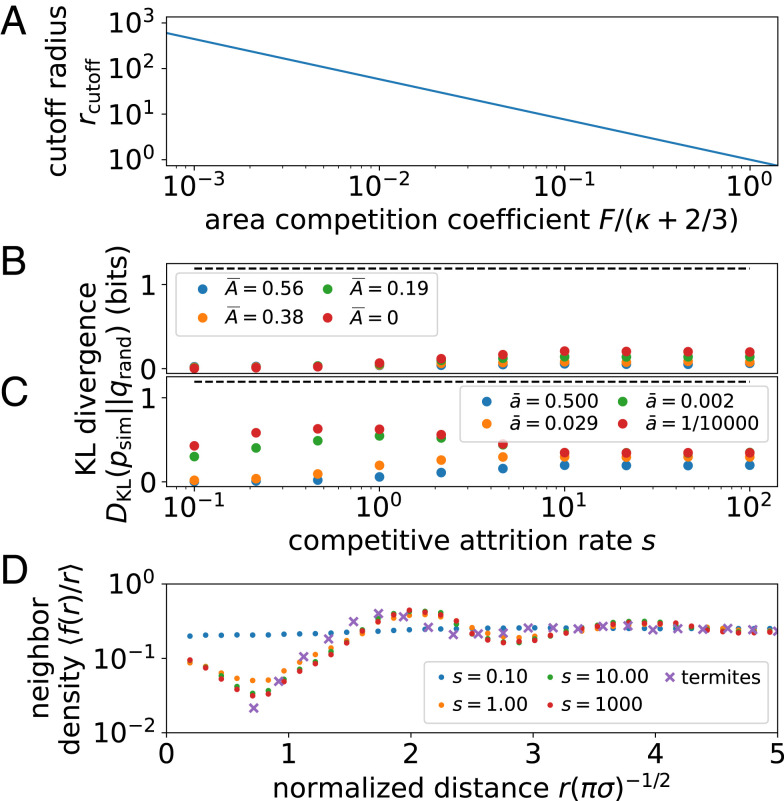
Characterizing trajectories through realm of models. (*A*) Location of cutoff to population number n(r) decreases as competitive costs increase, $r_{cuto\mathrm{ff}}\equiv {\left[F/\left(\kappa +2/3\right)\right]}^{-1/\left(\kappa +2/3\right)}$, tracing the gray cylinder in Fig. 3. (*B* and *C*) Deviations from randomness indicate emergence of order measured by Kullback-Leibler (KL) divergence of the nearest-neighbor distance distribution for simulation $p\left(r_{\text{min}}\right)$ from random points $q\left(r_{\text{min}}\right)$. *B* shows randomness-dominated phase when growth rate is significant, $3c_{m}^{2/3}ā/8=0.3$, and mortality rate Ā is small, while competitive attrition rate s varies. *C* shows signs of ordering when mortality rate is negligible Ā=0, while growth rate ā is driven to zero. Around s=1, we find a “liquid” phase, where organisms are densely packed but without long-range order. Dashed black lines indicate KL divergence measured at the “solid” phase. Bin size is set to Δr=1/20. (*D*) Normalized neighbor density at distance r, <f(r)/r>, indicates solid, hexagonally packed phase. Function f(r) counts all neighbors at distance r with f(0)=1 and is plotted against distance normalized by average spacing $1/\sqrt{\pi \sigma }$ given density σ. For comparison, we show the neighbor density for Namibian termite mounds from ref. 18, which are more tightly packed than in our simulation. Small s corresponds to the weak attrition limit, where the spatial distribution is nearly random.

Moving across the gray cylinder extending out from WEB theory in Fig. 3*A* corresponds to strengthening competitive interactions by increasing competitive attrition rate s. This region describes a set of models with a fixed population scaling exponent but with cutoffs in population scaling changing according to Eq. **12** and shown in Fig. 4*A*. Such effects can obscure scaling laws. As we show in Fig. 4*B*, however, strong variation in population number is not reflected in the statistics of nearest-neighbor separation until metabolic processes are severely suppressed. Fixing growth rate to $3c_{m}^{2/3}ā/8=0.3$ and varying the death rate, Ā, we find that the nearest-neighbor distribution hardly changes. However, after we fix Ā=0 and drive growth rate to zero, we begin to see the emergence of a different phase in Fig. 4*C*. In this limit and for moderate competitive attrition m, the system condenses into a disordered packing or liquid-like phase (*SI Appendix*, Fig. S4). Nevertheless, long-range order fails to appear because nearest-neighbor statistics are dominated by disorder introduced by turnover from randomly placed seedlings and continuously changing tree size. In other words, metabolic growth and death act on sufficiently fast timescales that regular patterns in spacing take too long to stabilize. In organisms with different rules for metabolic scaling, we may expect to find stronger tendencies for self-organization.

Such an example manifests in the semiregular packing of the fairy circles shown in Fig. 1. Such spacing entails a relatively narrow and peaked distribution of mound areas at some maximum size, a phenomenon incompatible with scale-free growth. Instead, this distribution implies that mounds that approach the maximum size are stable and that strong competitive interactions inhibit the formation of new smaller mounds. We can approximate such dynamics by driving growth and natural mortality to zero and vastly enhancing competitive mortality. This ensures that mounds are fixed at a typical size, with rigid boundaries delineated by strong competitive interactions and close to hexagonal spacing that minimizes survival of randomly placed colony seeds.^¶^ As we draw in black dashed lines across Fig. 4 *B* and *C*, the simultaneous limits of slow growth (ā→0), slow death (Ā→0), and lethal competition (s→∞) return large values of the KL divergence relative to random (*SI Appendix*, Fig. S5). As a more direct check, we show that the density of neighbors oscillates (Fig. 4*D*), analogous to fairy circle data from ref. 18 for which several mechanisms have been proposed. This is not the case for the disordered packing regime, where local exclusion is important but does not lead to long-range order. Thus, hexagonal packing is confined to a tight region of parameter space in our metabolic growth framework where growth is bounded (*SI Appendix*, section D). This region corresponds to a wide separation of timescales: Growth must be sufficiently slow to avoid introducing spatial disorder on the timescales with which relatively fast competitive death stabilizes regular spatial patterning (8, 73).

## 4. Transient Dynamics and Population Shock Waves

**Fig. 5.**
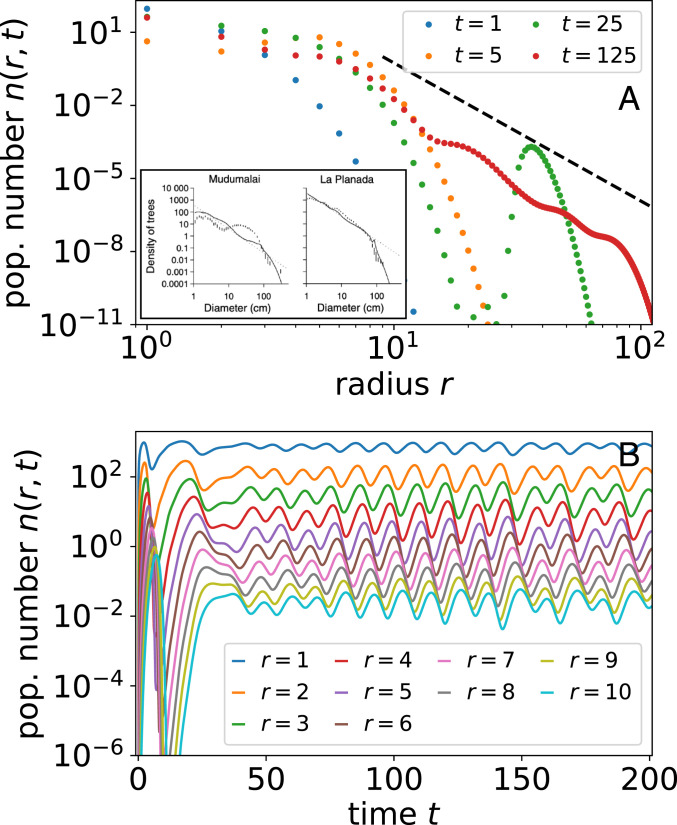
Oscillations in population number n(r,t) from asymmetric competition such as canopy cover. (*A*) Population number distribution n(r,t) at different times. *Inset* shows data from two tropical forests from ref. 32, where markers correspond to data and lines correspond to their model. Dashed black line shows predicted slope at steady state from Eq. **4**. (*B*) Population number oscillations for trees of different sizes. *SI Appendix*, Fig. S6 shows examples of oscillations in 2D automaton simulation. We use the Heaviside theta function for $1-\Lambda \left(r^{\prime }-r\right)=\Theta \left(r^{\prime }-r-\Delta r_{crit}\right)$.

Demographic stability among a population of sessile organisms is not guaranteed since ecosystems are buffeted by a wide range of endogenous and exogenous perturbations (20, 60, 74). For example, local competition is negligible in a young plot until individuals reach a size where they impinge upon neighbors, a phenomenon known as self-thinning (75). This dynamic is especially prominent in agriculture, where spacing is regular, plants are genetically identical, and competition onset is almost uniform (6, 7). Natural stands also vary with plot age, but they are more stochastic, and height differences can be prominent (34, 39). Remarkably in previous measurements, we find oscillations in population number with radius as depicted in Fig. 5 *A*, *Inset* taken from ref. 39, suggesting the presence of long-lived transience not captured by the steady state. Inspired by this observation, we consider how competitive asymmetry, specifically forces that increase mortality of smaller organisms, could excite population waves.

Asymmetric competition takes various forms such as how canopy shading reduces light incident on shorter plants lying underneath or around the larger ones with little cost to the latter (35, 43, 76). Large termite colonies are much more likely to destroy incipient colonies adjacent to their borders than face a threat (8). Susceptibility to exogenous disturbances like wind also depends on size, although sometimes to the benefit of smaller individuals (77, 78). As with symmetric competition, we formulate asymmetric competition in terms of its effects on population growth $\partial_{t}n\left(r,t\right)$. A mean-field framework means that the rate of decrease in population number is proportional to typical overlap between trees of radius r with all sizes larger than it up to $r_{\text{max}}$:

$$n\left(r,t\right)\;a_{can}\left(r\right)\int_{r}^{r_{max}}n\left(r^{\prime },t\right)\;a_{can}\left(r^{\prime }\right)\left[1-\Lambda \left(r^{\prime }-r\right)\right]\;dr^{\prime }.$$ [15]

We assume that the competitive effect $\Lambda \left(r^{\prime }-r\right)$ is some sigmoid-like function that decays from Λ(0)=1 to the limit Λ(∞)=0 (figure 4 in ref. 49) at which point the tallest trees completely obscure all light incident on ground area spanned by the canopy $a_{can}\left(r\right)=c_{can}r^{2\alpha_{can}}$, where $\alpha_{can}=2$ (33). A sigmoidal form indicates some characteristic length scale for Λ such that when the distance $r^{\prime }-r$ reaches some critical value $\Delta r_{crit}$, a substantial portion of light is obscured from above. This is distinct from symmetric interactions that scale with radius r and lack a typical length scale distinguishing competitors from noncompetitors. Thus, we consider asymmetric competition that is area delimited and favors larger organisms. This assumption is analogous to canopy shading and more generally captures the competitive advantage of larger organisms (36, 79).

As we show in Fig. 5*B*, canopy competition is negligible during initial forest growth but matters strongly when tall trees reach some critical density at which point the difference between the height of the tallest trees and the shortest ones is $\Delta r_{crit}$. At this point, smaller trees are at a sudden competitive disadvantage. Since the maximum tree cutoff is sharp in the population number n(r,t), the appearance of sufficiently large trees for light competition to matter is sudden and causes a correspondingly sharp die-off in young trees or a population shock. Likewise, the population number of small trees in Fig. 5*A* displays a sudden dip at short times. This dip in population number slowly propagates up to larger trees with growth. Eventually, canopy cover dips, and small tree population increases suddenly. The delay generates size-class oscillations that may be prominent when competitive interactions are strong and competition as a function of size difference is sharp. Linear stability analysis of the mean-field model suggests that oscillations may be a generic feature of competition between sizes (*SI Appendix*, section H). In the case of nonlinear metabolic growth, the rightward movement of waveforms in Fig. 5*B* accelerates with age: Radius grows superlinearly with time $r\left(t\right)\propto t^{3/2}$ when metabolic growth exponent b=1/3. Other than from superlinear growth, population waves also disperse because of stochasticity in tree growth. Such variation in shape and speed of population shock waves could be used to infer stochasticity in growth and competitive effects following endogenous or exogenous perturbation (60, 73).

Remarkably, we find oscillations in population number curves across data on tropical forests. Fig. 5 *A*, *Inset* displays two examples from ref. 32. Similar oscillations are visible across other tropical forests. For the example from La Planada, the widths of undulations seem consistent with an example from our simulation in Fig. 5*A*, although this is not the case for Mudumalai, which shows intriguingly wide oscillations. An unanswered question is whether such oscillations are internally generated from tuning of competitive parameters or originate from widespread and repeated exogenous perturbations (74). After all, competitive dynamics between organisms at different points of maturity can generate oscillatory cycles perhaps influenced by or affecting other classic ecological dynamics (25, 58). Although qualitatively similar curves in demographic data are presented as evidence against metabolic scaling—indeed, space filling yields poor explanation—our model shows that such deviations may arise due to dynamics overlaying metabolic scaling.

When considering asymmetric competition with resource fluctuations, we find important differences from symmetric competition. Resource constraints impose a limiting cutoff in maximum size and dampen population waves. As in the case of symmetric competition, accounting for resource constraints introduces an exponentially decaying tail that dominates near the point where resource limitations delimit the largest sustainable size. When resource limitations are sufficiently weak that there exists a wide scaling region in the population number that goes as $n\left(r\right)∼r^{-\alpha }$, asymmetric competition fixes the population number exponent to (*SI Appendix*, section G)

$$\alpha =4\alpha_{can}+2-b.$$ [16]

In contrast with Eq. **7**, Eq. **16** is free of metabolic growth coefficients but depends on the way that canopy area grows with radius, $2\alpha_{can}$, and metabolic growth exponent b. Thus, asymmetric resource competition leads to a different form for scaling exponent α than that of canonical metabolic scaling theory, its value generally incompatible with α=2 because of physical limits on values of $\alpha_{can}$. Although population oscillations likely share exogenous origins, it is remarkable that competition dynamics, although discussed widely in the literature (7, 71), present one endogenous cause, whose dynamical consequences are hardly remarked upon and suggestively aligned with data.

## 5. Discussion

The physical structure of a tree is a beautiful fractal not only along its visible constituents, trunk to branches to twigs, but down to the microscopic vasculature that shuttles products of photosynthesis from its self-similar canopy to a branching network of roots. It is remarkable then that even groups of trees seem to obey this pervasive fractal law such that the trees of a particular size “branch off” into trees of a smaller size and so on in such a way that we can consider, over some range, the set of large trees as a scaled set of smaller trees (34). This self-similar structure, reflected in power law scaling, emerges from consideration of energetic constraints translated into the requirement that trees fill the available canopy space (33). Yet, other sessile organisms fill space in a variety of ways determined by analogous mechanisms of growth, death, and competition (8, 62). Inspired by the forest picture, we propose a minimal model of sessile organism growth incorporating aspects of allometric scaling theory and area-based competition. From these basic principles, we obtain a general framework for competitive forces driven by metabolic requirements and fluctuating resources. When interaction with the environment dampens resource fluctuations (e.g., niche construction) or changes competitive interactions (e.g., symbiosis), these perturbations will be reflected in the spatial distribution of organisms (8) (Fig. 1). In this sense, the spatial distribution may serve as an indicator not only of changing conditions but also of how competition evolves in altered environments (18, 60).

We explore such variation by tuning competitive forces in our model with a tractable mean-field theory that succinctly relates metabolic and competitive effects in exponent relations. In the context of resource-area competition, competitive effects are most prominent in the population statistics of the largest organisms. This is because area-delimited competition must scale superlinearly with radius such that it dominates for the largest organisms (Eq. **11**). In comparison, individual metabolic growth and mortality scale sublinearly (41), indicating two different regimes of population number for symmetric competition: individual-dominated scaling and competition-mediated cutoffs (Fig. 2). Asymmetric competition, however, exacts a toll in a scale-free way because relatively larger competitors grow, die, and compete the same at every level. Then, competition is manifest in the population number exponent (Eq. **4**), affecting both scaling and cutoffs. Our formulation of competitive interactions establishes a basis to be extended to capture environment- or organism-specific variation in resource stress response or sharing such as by incorporating a distribution of diverse allometries. Yet, it also highlights how such diversity maps to universal features summarized by exponents that quantitatively link environmental fluctuations and metabolic scaling (Eq. **12**). Although real systems are noisy, finite, and constantly perturbed, our idealized assumptions about allometries serve as a way to organize models incorporating many more realistic features, a mapping that can be made exact by taking the limit to large systems (80, 81).

Other than indicating limitations of metabolic scaling theory—namely that it may be more accurate in forests with weaker local competition and smaller environmental fluctuations—our work suggests limitations of spatial correlation-based measures of regularity when varying organism size introduces disordered spacing (2). As we show by comparing the form of the nearest-neighbor distance distribution with the KL divergence (Eq. **13**), this measure changes weakly with competitive strength, suggesting that statistical approaches to measuring competition are limited. Instead, an integrated framework considering deviations from predicted scaling in demographics as well as spatial patterning may better specify the range of competitive forces acting across environments (4, 18, 60, 82, 83).

Beyond competitive forces, we find strong additional constraints necessary to stabilize spatial order in models with metabolic growth (Fig. 3). Whereas metabolic scaling tends to inject spatial disorder by constantly changing organism size and by opening free space upon organism death, regular tiling such as seen for fairy circles and some termite mounds requires the elimination of unbridled growth along with slow natural mortality and overwhelming competitive attrition in a background of sparse newcomers. This emergence of order starting with individuals and their metabolic dynamics presents a complementary perspective to field-theoretic derivations of vegetation patterns that start instead with densities (17, 84–86). In a similar vein, some field-theoretic models, such as those introduced by Martínez-García et al. (87, 88), have incorporated competition with local (logistic) limits on growth. That hexagonal packing occurs only in a corner of the much broader model space encompassed by our theory (Fig. 3) reflects the extraordinary nature of such regular patterns.

Complementary to the connection between spatial patterns and asymmetric competition (79, 89), we explore transient dynamics in the context of a size-based competitive hierarchy (58). Asymmetric competition can couple different timescales to one another and lead to oscillatory modes in size classes and thus, population number (Fig. 5)—although touching on the topic of self-thinning, our model extends beyond the typical focus on monoculture stands (6, 7, 71, 75). When there is a threshold at which such effects become important, such as with canopy light competition, we expect to find similar population shock waves. Remarkably, oscillatory modes manifest in multiple datasets of tropical forest demography (39). Such die-offs also may be observable in other systems or directly measurable if future data collection permits highly resolved temporal data on organism death. Furthermore, the lifetimes of these transient phenomena, indicated by width evolution, may allow us to distinguish internal competitive forces by leveraging demographic perturbation (20, 90). Oscillatory modes and instabilities are a widely studied feature of biological populations [for example, with the classic logistic equation (91, 92)], and metabolic growth in sessile organisms presents an unexplored mechanism by which they can arise.

Growth, death, and competition are essential characteristics of life. Yet, for particular ecosystems, assumed allometric forms for these forces will need to be refined. More specifically, we assume that demographic diversity is summarized in terms of individual age, which then determines individual properties like size and growth rate. Notably, the relations we derive do not have to be fixed by principles like space filling as is assumed for metabolic scaling of forests (33), and so, they relay a universe of possible allometries and environments. Nevertheless, power law scaling relations are an approximation of a population, and individuals will deviate from them (42). Accounting for such variation may be important in high-resolution models (93, 94). More fundamentally, organism growth or death may not neatly abide by allometric scaling relations with respect to age. We show one extreme in the example of hexagonal packing, where growth is truncated. Although technically, this is still a form of allometric scaling (if a trivial one), the analogy is only mathematically exact in the limit where individuals live for a long time, and thus, the growth period is short. Individual organisms that do not obey such behavior, perhaps displaying persistent oscillations in size, would present a new set of dynamics extending beyond the canonical picture of metabolic growth. We emphasize that such extensions would be natural and interesting to incorporate into the framework we present here.

The most striking ecological patterns occur when local interactions generate large-scale regularities, propagating information coherently over large scales and long times (95, 96). Fairy circles and termite mounds are a breathtaking example. Although forests, fairy circles, and termite mounds all seem to obey forces driving the cycle of birth, growth, and death at the level of the individual, population-level structure varies widely. Even among forests, some are characterized by randomly spaced trees, such as the examples we show here, but others, such as the pinyon–juniper ecosystem of the US Southwest, are more spatially regular. To connect the wide range of spatial patterns shaped by competitive forces in sessile organisms, we build on theoretical foundations of metabolic scaling. The resulting realm of models may frame analogies between organisms across species, environments, and times in the language of competitive forces acting on top of individual properties constrained by metabolic principles.

Previously published data were used for this work from Tschinkel (1), Grohmann et al. (2), Muvengwi et al. (3), Adams and Tschinkel (4), and Schneider et al. (5).

## Supplementary Material

Supplementary File

## Data Availability

Code for reproducing the results shown in this work is available at https://github.com/eltrompetero/simple_sessile.

## Footnotes

*In the context of water uptake, $\eta_{1}\approx 1.8$ (49).

^†^When ν≤2, we must also fix an upper limit to the distribution to ensure a finite mean.

^‡^In the marginal case $\eta_{1}=2\alpha_{r}$, the coefficient of competition modifies the scaling exponent α. This is a mathematical possibility but unrealistic.

^§^Figure 4 in ref. 70 shows termites in several soil types, and *SI Appendix*, Fig. S8 shows variation among plots in Alaskan rainforest.

^¶^In the “zero-temperature” limit where competitive mortality always selects out the weaker of two competitors, it is clear that tight packing is stable to disorder because any newcomer must overlap with more than one other individual, whereas every individual forming the lattice only intersects with the newcomer. Hexagonal packing is the densest of packings and thus, most stable to infiltration.
