# Supplementary material for: Growth, death, and resource competition in sessile organisms
Source: Proc Natl Acad Sci U S A. 2021 Apr 9;118(15):e2020424118. doi: 10.1073/pnas.2020424118 (PMC8053998; doi:10.1073/pnas.2020424118)
Supplement: Supplementary File [file pnas.2020424118.sapp.pdf]

Supplementary information: Growth, death, and resource competition in sessile organisms

Edward D. Lee[a], **Christopher P. Kempes**[a], **and Geoffrey B. West**[a]

[a]Santa Fe Institute, 1399 Hyde Park Rd, Santa Fe, NM 87501

This manuscript was compiled on March 24, 2021

A. From discrete to continuum size-class model

Though tree population is typically binned into discrete size classes in both observation and theory, tree growth is in reality a function of a continuous radius r. In the main text, we show discrete and continuum limits in Eqs 3 and 4. To go from one to the other, we relate the index k to radius r_k such that $r_k \equiv r_0 + k\,\Delta r$. Then, taking Eq 3 and Taylor expanding the growth rate in terms of Δr, we obtain $\dot{r}(r+\Delta r) \approx \dot{r}(r) + \Delta r \partial_r \dot{r}(r) + \mathcal{O}(\Delta r^2)$, where the last term contains all terms of quadratic and higher order. Likewise, we expand population number $n(r,t)$ about r. After rearranging terms, we obtain

$$\partial_t n(r,t) = -\Delta r \partial_r [n(r,t)\dot{r}(r)/\Delta r] - n(r,t)\mu(r) + \mathcal{O}(\Delta r^2). \quad \text{[S1]}$$

In the limit $\Delta r \to 0$, we can discard the second- and higher-order terms $\mathcal{O}(\Delta r^2)$ to find Eq 4. In Eq S1, we have made explicit the dependence on bin width, which arises from our definition of growth rate. In this formulation, the number of trees located within the range $[r, r+\Delta r)$ that will grow to the next size class in the time interval dt is

$$n(r,t)\dot{r}(r)dt/\Delta r, \quad \text{[S2]}$$

where we must ensure that the "distance" grown $\dot{r}(r)dt$ after a small time step dt is smaller than Δr to assure that growth rate does not change meaningfully within the bin and that trees do not simply pass through a bin in numerical simulation. We must take special care with this limit in the boundary condition for saplings in Eq 5. As a result, such discreteness is essential for relating coefficients of growth and mortality with their measurements from observational data.

With these equations in hand, we study what happens at steady state by setting the time derivative $\partial_t n(r,t) = 0$,

$$0 = -\dot{r}(r)\partial_r n(r) - n(r)\partial_r \dot{r}(r) - n(r)\mu(r). \quad \text{[S3]}$$

After rearranging terms, we find

$$\frac{\partial_r n(r)}{n(r)} = \frac{-\partial_r \dot{r}(r) - \mu(r)}{\dot{r}(r)}. \quad \text{[S4]}$$

This can be integrated directly after recognizing the left-hand side to be the derivative $\partial_r \log n(r)$. Then, the general solution without having specified the functional forms for growth and death is

$$n(r) = n(r_0)\exp\left(-\int_{r_0}^{r} \frac{\partial_{r'}\dot{r}(r') + \mu(r')}{\dot{r}(r')}\,dr'\right). \quad \text{[S5]}$$

In other words, the steady-state population number depends on the balance of growth-rate curvature and mortality over growth rate, determining the total amount of incoming flux as is explicitly solved using metabolic scaling theory in the main text.

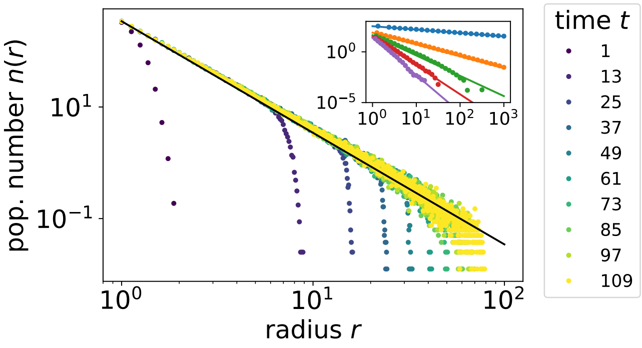

Fig. S1. Transience in compartment model starting with empty plot from automaton simulation ($\alpha = 2$, $g_0 = 10^3$ trees per unit time). (inset) Steady-state profiles for varying scaling exponent α (Eq 7). Discreteness of bins slightly flattens the slope for small radius r.

Starting with an empty plot, we expect to find transient behavior as individuals grow and fill the available space which must violate steady-state predictions. In the case of the simple metabolic scaling compartment model, the transient is uncomplicated: small trees quickly approach the steady-state scaling form $n(r) \sim r^{-\alpha}$ and a sharp cutoff moves to the right as in Figure S1, where we plot the results of a stochastic automata simulation in a two-dimensional box. When forests are still growing, we would expect such a cutoff to be prominent and even obscure the scaling form. Thus, it is crucial to consider the age of the plot before comparing with steady-state assumptions as is discussed in references (1–3).

B. Metabolic scaling theory

In Eq 1, we consider an approximation derived from metabolic scaling laws relating radial growth rate in trees with current size measured by radius. This equation derives from the allometric scaling prediction relating biomass growth rate $\dot{m} = \bar{a}m^{3/4} - \bar{b}m$ such that quarter-power scaling only dominates for modestly sized organisms. The exact transition point where quarter-power scaling becomes a poor approximation depends on the ratio of parameters for biological energetics $\bar{a}/\bar{b}$. For interspecies scaling over a large range, however, data shows the linear term to be negligible, or $\bar{a}/\bar{b} \gg 1$ (4). In reference (5), entire trees are quite literally plucked and analyzed to confirm that this quarter-power scaling holds once trees are of mass $m = 0.1\,\text{kg}$, which constitute the "saplings" we consider. When we use the scaling relation $r = c_m m^{3/8}$ to go from mass to radius, we neglect these corrections and hence the

[2]To whom correspondence should be addressed. E-mail: edlee@santafe.edu

approximation sign in Eq 1.

C. Mean-field theory of symmetric competition

Here, we discuss in deeper detail the derivation of the mean-field theory for symmetric resource competition. In the main text, we focus on the example of tree mortality rate as a function of overlapping root area with neighbors.

Starting with Eq 8 defining metabolic inequality ΔQ, a quantity that must be positive to guarantee survival, we picture placing a tree randomly on the plot and asking with what probability it lands on a region already covered by other trees. Averaging over many spatial arrangements over a long period of time, we can consider the competitive force exerted by others to constitute a kind of "mean-field,"

$$\Delta Q = \varepsilon\rho(t)a(r)\left[1 - f\frac{A_{\mathrm{tot}} - a(r)}{L^2}\right] - Q_0(r), \qquad \text{[S6]}$$

where $A_{\mathrm{tot}} \equiv \int_{r_0}^{r_{\max}} n(r')a(r')\,dr' \propto r_{\max}^{1+2\alpha_r}/(1-\alpha+2\alpha_r)$ is the total area covered by all individuals, where an individual of radius r' covers area $a(r')$, for a square plot of linear dimension L. As in the main text, we denote resource extraction efficient ε, time-fluctuating resource density $\rho(t)$, resource sharing fraction f, and basal metabolic requirements $Q_0(r)$. Eq S6 accounts for the probability of landing on area occupied by other trees is the simply the fraction of the plot that is covered assuming that trees are not overlapping. This is a key assumption, and in the main text we further assume that the typical ratio of occupied tree area to the plot area is unity, $A_{\mathrm{tot}} \approx L^2$, or that all available space is filled. This is clearly a poor approximation when there is much overlap and $A_{\mathrm{tot}} \gg L^2$ or when the plot is sparse such that $A_{\mathrm{tot}} \ll L^2$. However, this dependence ends up only determining the location of the maximum tree size cutoff and not the form of the exponential tail given by the exponent κ.

Given this major simplification that leads to Eq 9, the probability of mortality is determined by the probability that incoming resources are insufficient to cover basal metabolic rate, what we call the "probability of fatal fluctuations,"

$$\begin{aligned} p\,(\xi > \xi_{\mathrm{basal}}) &= \int_{\xi_{\mathrm{basal}}}^{\infty} h(\xi')\,d\xi' \\ &= \xi_0^{\nu-1}\int_{\xi_{\mathrm{basal}}}^{\infty} \xi'^{-\nu}\,d\xi' \\ &= \xi_0^{\nu-1}\frac{1}{1-\nu}\left[\xi^{1-\nu}\right]_{\xi_{\mathrm{basal}}}^{\infty} \end{aligned} \qquad \text{[S7]}$$

Assuming that $\nu > 1$,

$$p\,(\xi > \xi_{\mathrm{basal}}) = \frac{(\xi_{\mathrm{basal}}/\xi_0)^{1-\nu}}{\nu - 1}, \qquad \text{[S8]}$$

where ξ_0 is chosen to enforce that the average $\bar{\xi} = 1$. When $\nu \leq 1$, the integral diverges with the upper cutoff, and it must be explicitly specified. The upper cutoff must also be specified to calculate a finite mean for $1 < \nu < 2$, which emphasizes the importance of large fluctuations when ν is small. Though we primarily refer to the power-law scaling form as a reduced representation of the propensity for large resource fluctuations, rainfall has been shown to display fluctuations suggestive of self-organized criticality (6). When we measure

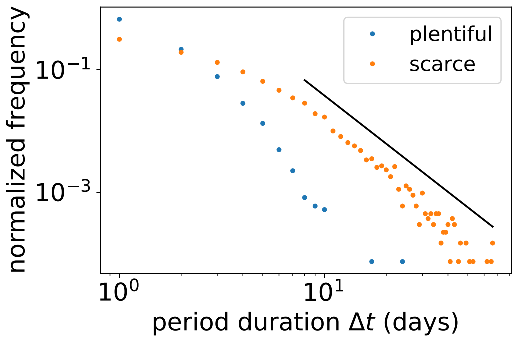

Fig. S2. Histogram of periods with above- and below-average rainfall, labeled as "plentiful" and "scarce," respectively, in weather stations across Puerto Rico from early 1970s to mid 2010s. To guide the eye, we have included a power law tail $\Delta t^{-2.6}$ as a black line.

rainfall at NOAA weather stations across Puerto Rico, we find that durations of periods showing below average rainfall display a power-law-like tail in Figure S2 (7). Nevertheless, we emphasize that the principal role of the power-law form is to clearly distinguish between scenarios where large fluctuations are negligible ($\nu > 2$) from when they are not ($\nu < 2$). The special case of $\nu = 2$ corresponds to Zipf's law, where the scale of fluctuations, i.e. the variance, depends on the scale of observation.

We determine ξ_{basal} by asserting that the metabolic rate inequality in Eq 9 is satisfied, leading to

$$\frac{1}{\xi_{\mathrm{basal}}} = \frac{Q_0(r)}{\varepsilon\bar{\rho}\,a(r)[1-f]}. \qquad \text{[S9]}$$

Now, using allometric scaling relations $a(r) = c_{\mathrm{r}} r^{2\alpha_{\mathrm{r}}}$,

$$\begin{aligned} p(\xi > \xi_{\mathrm{basal}}) &= \frac{1}{\nu-1}\left(\frac{\beta_1\xi_0 r^{\eta_1}}{\varepsilon\bar{\rho}\,c_{\mathrm{r}}(1-f)r^{2\alpha_{\mathrm{r}}}}\right)^{\nu-1} \\ &= \frac{1}{\nu-1}\left(\frac{\beta_1\xi_0}{\varepsilon\bar{\rho}\,c_{\mathrm{r}}(1-f)}\right)^{\nu-1} r^{(\nu-1)(\eta_1-2\alpha_{\mathrm{r}})} \end{aligned} \qquad \text{[S10]}$$

where $\xi_0 = (\nu-2)^{1/(2-\nu)}$ to fix the average $\bar{\xi} = 1$ assuming $\nu > 2$. In the main text, we recast Eq S10 as Eq 10 defining exponent κ and coefficient B. Though the full analytic expression for B is in Eq S10, we also recognize that the probability of death is assured when organisms surpass basal requirements, or $p(\xi > \xi_{\mathrm{basal}}) \to 1$. This leads to the expression for B in the main text, which is determined by $\bar{\rho}$, or typical resource density.

Then, we solve for the steady-state solution by including into Eq S5 an additional term for resource deprivation, $Bs\,r^{\kappa}$, as in Eq 11. Given $\kappa > b$ and integrating yields

$$\log\left(\frac{n(r)}{n(r_0)}\right) = -\left(b + \frac{8\bar{A}}{3\bar{a}c_m^{1-b}}\right)\log\left(\frac{r}{r_0}\right) - \frac{8\,s\,c_m^{b-1}}{3\bar{a}(\kappa+1-b)}B\left(r^{\kappa+1-b} - r_0^{\kappa+1-b}\right). \qquad \text{[S11]}$$

Exponentiation of both sides gives Eq 12, where the constants have been absorbed into the normalization constant $\tilde{n}_0$.

Though symmetric competition does not in principle change metabolic scaling, a sufficiently narrow scaling regime (or correspondingly strong enough of a tail) could mask scaling, effectively nullifying the space-filling assumption of metabolic scaling. In particular, it may be difficult to distinguish metabolic scaling from metabolic-scaling-like tails with limited range of observation such as in the examples shown in reference (8).

D. Beyond power-law scaling

The framework that we propose in the previous sections for a demographic theory incorporating growth, death, and resource competition can be naturally extended to alternative organism demographics, namely forms beyond the power laws that we consider in the main text. As an example of such an extension, we consider logistic growth, exponentially decaying mortality rate, and area-based resource competition. Though the particular nature of such functions matter for determining the full form of the population number distribution $n(r)$, we also show that truncated growth generally allows for a sudden transition from a population consisting of mostly small organisms to one mostly of the largest organisms.

We consider a logistic growth function as an example of bounded growth, where the radius r of the sessile organism changes with time t as

$$r(t) = \frac{r_{\max}}{2}\left\{\tanh[\alpha(t-t_{1/2})]+1\right\}. \qquad \text{[S12]}$$

Eq S12 refers to maximum radius $r_{\max}$, inverse growth duration α, and time at which organism reaches half its maximum size $t_{1/2}$. We replace the variable t in favor of r by first taking the time derivative,

$$\dot{r}(r) = \frac{r_{\max}\alpha}{2}\,\mathrm{sech}[\alpha(t-t_{1/2})]^2,$$

then the length derivative,

$$\partial_r \dot{r}(r) = -r_{\max}\alpha^2\,\mathrm{sech}[\alpha(t-t_{1/2})]^2\tanh[\alpha(t-t_{1/2})]\frac{\partial t}{\partial r}.$$

Substituting in r for t from Eq S12,

$$\begin{aligned}\partial_r \dot{r}(r) &= -2\alpha\tanh[\alpha(t-t_{1/2})]\\ &= -2\alpha\left(\frac{2r}{r_{\max}}-1\right)\end{aligned} \qquad \text{[S13]}$$

This means that logistic growth is a simple linear decrease of growth rate as a function of radius r until maximum radius $r_{\max}$ has been reached.

As for mortality, we assume that it decays on some characteristic timescale τ_μ (i.e. it is not scale-free). We take for an example an exponential decay with time, $\mu(t) = \mu_0 e^{-t/\tau_\mu}$,which can also be expressed as a function of radius,

$$\mu(r) = \mu_0 e^{-t_{1/2}/\tau_\mu}\exp\left(-\frac{1}{\alpha\tau_\mu}\tanh^{-1}\left[\frac{2r}{r_{\max}}-1\right]\right). \qquad \text{[S14]}$$

As derive in the previous section, we take competition to be motivated by resource fluctuations and competition such that it goes as $B\,s\,r^\kappa$. Putting all of these together, we find that at steady state

$$n(r) = n(r_0)\exp\left(-\int_{r_0}^{r}\frac{\partial_{r'}\dot{r}' + \mu(r') + B\,s\,r'^\kappa}{\dot{r}'}\,dr'\right). \qquad \text{[S15]}$$

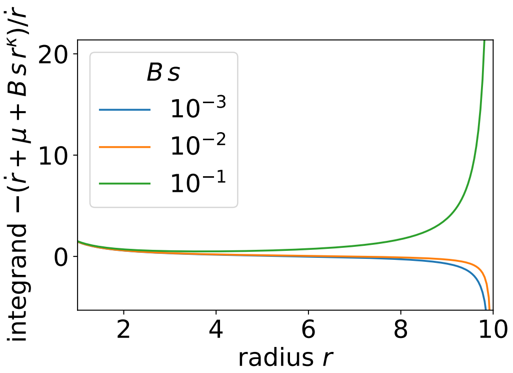

Fig. S3. Integrand of Eq S15 across range $r_0 = 1$ to $r_{\max} = 10$. Sign of the tail depends on the coefficients of death including both natural mortality and competition. Here, we change the coefficient of competition $B\,s$, which shifts the tail from a large negative number (few large organisms) to a large positive number (many large organisms). Parameter values are defined in the included code.

Unlike in the power law framework where the analogous form for Eq S15 can be solved with straightforward analytical approaches, it is not so straightforward to solve here.

Nevertheless, we can obtain some intuition about Eq S15 by considering the integrand, which will reveal how the competing forces trade off for organisms of different sizes. It is immediately clear that for a growth function that goes to 0 for finite $r_{\max}$, the integrand must diverge. If the sign of the divergence is positive, then $n(r_{\max}) = 0$, but if it is negative then the population must be dominated by the largest organisms, $n(r_{\max}) \to \infty$ as we show in Figure S3. By modulating the coefficients of the respective terms in the numerator of the integrand, it then becomes possible to switch between these two tail regimes. This switching in the tail is a generic feature that results from growth stopping for the largest organisms. In the example of the logistic function, growth does not truly stop, but it does approach 0 exponentially quickly with time, meaning that the integrand in Eq S15 explodes with radial growth. If growth slows dramatically enough, the particular details of how death and competition decay are not important: as long as they remain finite, then it is possible to obtain a transition between small-organism or large-organism dominated states by modulating the factors of death, either natural mortality or competition-induced mortality. Then, we find a sudden switch in demographics — the divergence implies that the switch will be very sudden with respect to a small change in the coefficients of the mortality terms — which aligns with the emergence of spatial order that we find in sessile populations with truncated power-law growth.

E. Estimating Kullback-Leibler divergence

We use the Kullback-Leibler (KL) divergence to compare the shape of the nearest-neighbor distance distribution in data or simulation $p(r)$ with a random null model $q(r)$. KL divergence represents a fundamental measure of distinguishability between two probability distributions (9), but there are subtleties in estimating information quantities with finite data (10). Namely, a bin width Δr must be chosen for the distance between neighbors r.

As defined in the main text and repeated here, the KL divergence between two distributions defined over distance r

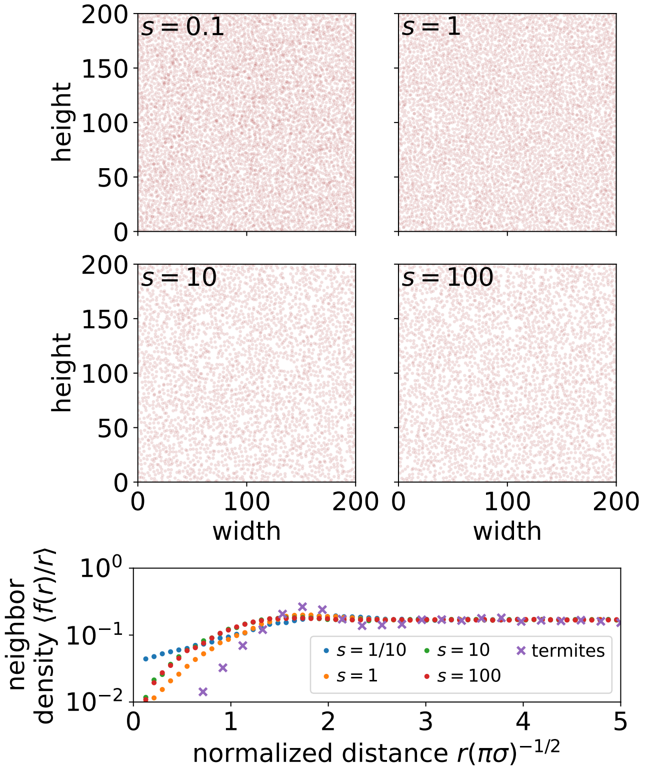

Fig. S4. (top) "Liquid" phase showing dense packing of individuals without long-range order. These plots are examples of ones on which we measure the KL divergence shown in Figure 4C. When competitive attrition rate s is too large, disordered dense packing is no longer possible, presaging the emergence of hexagonal packing with less random overlap. (bottom) Neighbor density function in disordered packing, or "liquid," phase. Unlike hexagonal packing as in Figure 3, there is no long-range ordering, but local exclusion is evident in the dip near $r = 0$.

is

$$D_{\mathrm{KL}}[p||q] = \int_{r_0}^{\infty} p(r) \log\left(\frac{p(r)}{q(r)}\right) dr. \qquad \text{[S16]}$$

In the case considered here, we have the analytic form for $q(r)$ in Eq 14 that accounts for first-order corrections for a finite plot in our simulation. On the other hand, we only have a statistical, sampled approximation to $p(r)$, the estimate from binning, $\hat{p}(r) = p(r)\Delta r + \epsilon(r, \Delta r, K)$, with error term ϵ that depends on radius r, bin width Δr, and sample size K. The corresponding linear approximation of the null distribution is $q(r_{\mathrm{i}})\Delta r$. Thus, the estimated KL divergence is

$$D_{\mathrm{KL}}[p||q] \approx \sum_{\mathrm{i}} \hat{p}(r_{\mathrm{i}}) \log\left(\frac{\hat{p}(r_{\mathrm{i}})}{q(r_{\mathrm{i}})\Delta r}\right) \Delta r, \qquad \text{[S17]}$$

where the sum is over every unique discretized radius $r_{\mathrm{i}} = r_0 + \mathrm{i}\Delta r$ in the sample.* Using the series expansion $\log(1+x) \approx x - x^2/2 + \mathcal{O}(x^3)$ for $x \ll 1$, we obtain an expansion of the form

$$D_{\mathrm{KL}}[p||q] \approx \sum_{\mathrm{i}} p(r_{\mathrm{i}}) \left[\log\left(\frac{p(r_{\mathrm{i}})}{q(r_{\mathrm{i}})}\right) \Delta r + \frac{\epsilon'}{K} - \frac{\epsilon'^2}{2\Delta r K^2} + \mathcal{O}(K^{-3})\right]. \qquad \text{[S18]}$$

*This requires us to assume that $0 \log 0 = 0$, which is justified by continuity (11).

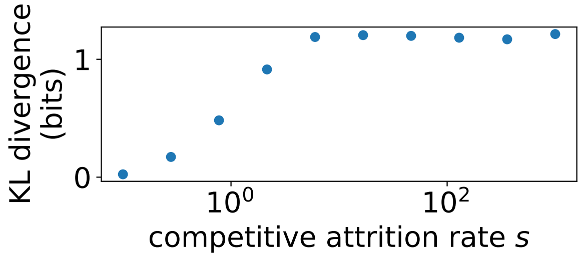

Fig. S5. KL divergence between simulation and random null model distributions of nearest-neighbor distance having set mortality rate coefficient $\bar{A} = 0$ and effectively set $\bar{a} = 0$ (growth follows a truncated power law). Only in this limit does hexagonal packing manifest as is indicated by the large divergence for $s \gg 1$. In order to simulate this limit, we set $\bar{A} = 0$ and allow incoming individuals to grow briefly before saturating at a maximum size. This procedure allows us to reach a stable arrangment much faster but corresponds to the same limit when this growth period is brief relative to all other timescales. To see an example of hexagonal packing in a plot of organisms, see Figure S9. Also see SI Section D for a consideration of population number function under the explicit assumption of logistic growth.

We group together all terms smaller than order K^{-3} in the last term. When we have a large sample, as we do for our simulations where $K \sim 10^5$, then the error terms are determined by finite sampling statistics and so have zero mean but variance that goes like $p(r_{\mathrm{i}})\Delta r[1 - p(r_{\mathrm{i}})\Delta r]/K$ as has been made explicit here by pulling out the K dependence in $\epsilon = \epsilon'/K$, defining the prime variable ϵ'. Barring the limit of minuscule bin size $\Delta r \sim K^{-1}$, where other corrections dominate, we heuristically choose a reasonably small bin size for the large sample set $\Delta r = 1/20$. Distinction of the regions of morphological phase space are robust to variation about this choice (given the previous conditions). Importantly, this allows us to track the emergence of disordered packing, the "liquid" phase, and the hexagonally ordered phase as we show in Figures 4 and S5, but a different series expansion may be required for comparison with smaller data sets and different plot boundary conditions.

F. 2D automaton model

At each time step of duration dt, three steps are taken in the following order.

1. All tree are grown into the next largest size class with rate given by the growth function $\dot{r}(r)/\Delta r$. Saplings are introduced into uniformly random locations of the plot at rate g_0.

2. Trees are removed from the system with probability given by mortality rate $\mu(r)$.

3. If root area competition is included, total resource available to each tree is calculated given a random state of the resource scarcity ξ. Tree overlap area Δa with all neighbors is calculated. Trees falling below the basal metabolic threshold are removed with a different rate $s\,\Delta a$.

4. If canopy competition is included, overlapping area Δa with taller (more than Δr_{crit} in height difference) trees is calculated. Trees are moved with rate $s'\Delta a$.

All variables including set parameters are listed in Table 1. As we argue in the main text, the properties of the simulation fall

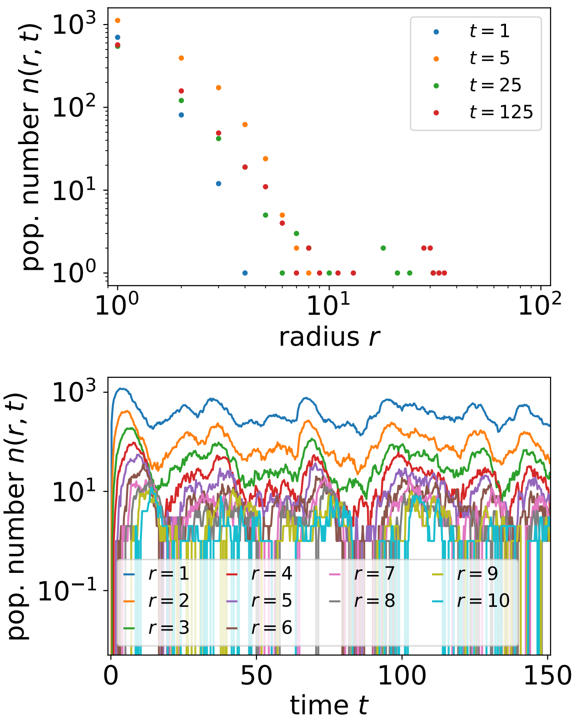

Fig. S6. Example of persistent population number oscillations for explicit 2D simulation like in Figure 5. For the automaton simulation of individual trees, stochasticity is important.

into several generic categories that rescale with the relationships between the considered timescales and dimensions. Note that the order of the steps commute in the limit the timestep $dt \to 0$.

In the context of transient growth, we set a small maximum tree size. Trees that reach this size cannot leave the size class by growth, only by death.

For particular model parameters and exact specification of the computational model, see the code repository at https://github.com/eltrompetero/simple_sessile. The code released with the paper is identified in file names with "PNAS".

G. Asymmetric size-competition exponent

We derive the scaling exponent for population number for resource competition with individuals larger than oneself. Using canopy competition as an example, the mean-field approximation dictates that averaged interaction with trees of the same or of larger size leads to competitive cost

$$n(r)a_{\mathrm{can}}(r)\int_r^\infty n(r')a_{\mathrm{can}}(r')[1-\Lambda(r'-r)]\,dr', \qquad \text{[S19]}$$

where the distance function $\Lambda(r'-r)$ indicates how strongly competitive effects come into play with difference in size. As in the main text, n is population number and a_{can} canopy cover area. We consider some function with a typical length scale Δr_{crit} which we approximate with the Heaviside theta function

$$1-\Lambda(r'-r) = \Theta(r'-r-\Delta r_{\mathrm{crit}}). \qquad \text{[S20]}$$

The key assumption is of a finite scale at which such effects become important, and the resulting exponent will not depend on whether or not Λ is something similar like a decaying exponential or a sigmoid, as is found in forests (2).

Now, we note that if it is the case that population number $n(r) = c_n r^{-\alpha}$ display a power-law tail and that canopy area scales with radius r as as $a_{\mathrm{can}}(r) = c_{\mathrm{can}} r^{2\alpha_{\mathrm{can}}}$, then the competitive cost is likewise scale-free. Integration of Eq S19 having substituted in Eq S20 yields

$$= n(r)\frac{c_n c_{\mathrm{can}}^2}{\alpha - 2\alpha_{\mathrm{can}} - 1} r^{4\alpha_{\mathrm{can}}+1-\alpha}. \qquad \text{[S21]}$$

This returns us to an analogous form of Eq 11 except now we have asymmetric competition instead of symmetric. At steady state, we have

$$\partial_r[n(r)\dot{r}(r)] = -n(r)\mu(r) - n(r)\frac{c_n c_{\mathrm{can}}^2}{\alpha-2\alpha_{\mathrm{can}}-1} r^{4\alpha_{\mathrm{can}}+1-\alpha},$$
$$\frac{\partial_r n(r)}{n(r)} = -\frac{\partial_r \dot{r}(r)}{\dot{r}(r)} - \frac{\mu(r)}{\dot{r}(r)} - \frac{c_n c_{\mathrm{can}}^2}{\alpha-2\alpha_{\mathrm{can}}-1}\frac{r^{4\alpha_{\mathrm{can}}+1-\alpha}}{\dot{r}(r)}. \qquad \text{[S22]}$$

First, we note that competitive interactions here must decay in a scale-free way for ever larger trees if the population number decays as a power law. This means that the last term in Eq S22 must be commensurate with the others.[†] If this is the case, then the scaling exponent must depend on asymmetric competition, implying that the last term goes as r^{-1} to match the scaling of the other terms under the assumptions of metabolic scaling theory, and thus we recover Eq 16. Unlike symmetric resource-area based competition, canopy-light competition only depends on the rate of canopy-area scaling and the metabolic-growth exponent and not the timescales of natural mortality and growth.

In principle, competitive dynamics could replicate WEB metabolic scaling if $\alpha = 2$ in Eq 16. However, this is unlikely because the resource area exponent is lower bounded $\alpha_{\mathrm{can}} > 1/2$ (otherwise it would not denote an area), which implies that the metabolic scaling exponent $b > 2$. Such superlinear growth is clearly at odds with observation which show sublinear scaling in biomass production not just in forests but across diverse biology (4, 12). This presents a prediction for the population scaling exponent that could be tested by considering environments where domination by larger organisms displays such scale-free behavior.

H. Stability analysis

We analyze the stability of our mean-field theory considering symmetric and asymmetric competition separately and find numerical evidence that instabilities are a generic feature of competitive interactions. This observation aligns with the intuition that population waves propagate through time because of growth and so any particular size population interacts with a delayed version of itself in the future. As is well known in dynamical control theory, population dynamics, and other physical models, self-coupling with time delays generally lead to oscillations and even chaos (13, 14).

To analyze the stability of our equations, we linearize them about the steady-state solution, denoted by $\bar{n}(r)$. We perturb

[†] Symmetric interactions, on the other hand, can be summarized as a constant mean-field effect that scales only with the area of the individual and not with population number.

Table 1. Parameters and variables reference table. As examples of how these parameters might be measured in real systems, we provide example units and a specific reference to the example of forests when applicable. For trees, natural units for the the radial length r may be the basal stem radius in centimeters, time t in weeks, and population count n across a plot of area commensurate with the total area covered $A_{\rm tot} = \int_{r_0}^{r_{\rm max}} A(r)\,dr$ if the largest tree is of radius $r_{\rm max}$. Metabolism could be measured by respiration rate such as moles per second (5), mass in kilograms, and birth and death rates in individual per week. For termite colonies such as $Macrotermes\ falciger$, we could instead measure the radial length in terms of radial width at the base of the mound with similar temporal and mass units since they align with the typical scale of termite mounds as well. More generally, appropriate measurement units would need to be set for respective organisms depending on what consistent and reasonable physical measurements can be repeated across many individuals. Such differences will change the coefficients between allometric properties but not the allometric exponents. In the main text, we do not specify particular units for our findings — as we point out here they will likely vary depending on the organism of interest — but the appropriate units can be determined using the definitions below.

parameter	description	units	forest example
$3c_m^{1-b}\bar{a}/8$	growth rate coefficient	[meters]$^{1-b}$[days]$^{-1}$	
a	symmetric competition area	[meters]^2	root area
$\bar{a}$	biological energetics constant for metabolic growth	[kilograms]$^{1/4}$[days]$^{-1}$	
$a_{\rm can}$	asymmetric competition area	[meters]^2	canopy area
A	area of plot	[meters]^2	forest plot
$A_{\rm tot}$	total area of all organisms in plot (double-counting overlap)	[meters]^2	all root area
$\bar{A}$	natural mortality coefficient	[individuals][day]$^{-1}$	
B	normalization coefficient for $p(\xi > \xi_{\rm basal})$	[meters]$^{-\kappa}$[individuals]$^{-1}$	
b	metabolic growth rate exponent $\dot{r} \sim r^b$, $b = 1/3$	unitless	
$c_{\rm can}$	canopy scaling coefficient	[meters]$^{2-2\alpha_{\rm can}}$	
c_m	coefficient relating mass scaling to radius $r = c_m m^{3/8}$	[meters][kilograms]$^{-3/8}$	
$c_{\rm r}$	root area scaling coefficient	[meters]$^{2-2\alpha_r}$	
$D_{\rm KL}$	Kullback-Leibler divergence	[bits]	
F	constant in length scale, see Eq 12, $8sBc_m^{b-1}/3$	[meters]$^{b-\kappa-1}$	
f	resource sharing fraction	unitless	$f = 1/2$ (zero-sum game)
g_0	new organism birth rate	[individuals][day]$^{-1}$	new seedling rate
$h(\xi)$	probability distribution of scarcity	unitless	
K	number of data points	unitless	
L	length of plot	[meters]	
m	organism mass	[kilograms]	biomass of tree
$n(r)$, $\bar{n}(r)$	population number at steady state	[individuals]	
$n(r,t)$	population number as a function of size and time	[individuals]	
$p(\xi > \xi_{\rm basal})$	cumulative distribution of scarcity above basal level	unitless	
Q	metabolic rate	[micromoles][seconds]$^{-1}$	respiratory output
$q(r_{\rm min})$	null random distribution for nearest neighbor distances	unitless	
r_0	smallest organism radius	[meters]	sapling basal stem radius
r_k	organism radius for size class k	[meters]	basal stem radius
$r_{\rm min}$	distance to nearest neighbor	[meters]	center-to-center distance
s	death rate when under resource stress	[individuals][day]$^{-1}$	
t	time	[days]	weeks
$u(r)$	perturbation function on top of steady state	[individuals]	
α	population number scaling exponent $n(r) \sim r^{-\alpha}$	unitless	
α_1	basal metabolic rate scaling exponent	unitless	
$\alpha_{\rm can}$	canopy radius scaling exponent with radius	unitless	
α_r	resource area scaling exponent with radius	unitless	
β_1	basal metabolic rate coefficient	[micromoles][meters]$^{-\eta_1}$[seconds]$^{-1}$	
Δa	overlap in area	[meters]^2	
Δr	size compartment bin width	[meters]	
ε	resource extraction efficiency	unitless	
η_1	resource area scaling exponent	unitless	$\eta_1 = 1.8$ (soil water usage)
κ	resource area competition exponent, see α_1 and ν	unitless	
Λ	lack of larger organism dominance	unitless	canopy light permittivity
μ_k	natural mortality rate for size class k	[individuals][day]$^{-1}$	
ν	resource fluctuation exponent	unitless	
ξ	scarcity	unitless	precipitation fluctuation
$\rho(t)$	resource density	[micromoles][seconds]$^{-1}$[meters]$^{-2}$	rainfall per unit area
$\bar{\rho}$	average resource density	[micromoles][seconds]$^{-1}$[meters]$^{-2}$	rainfall per unit area
σ	organism density	[individuals][meters]$^{-2}$	trees per unit area

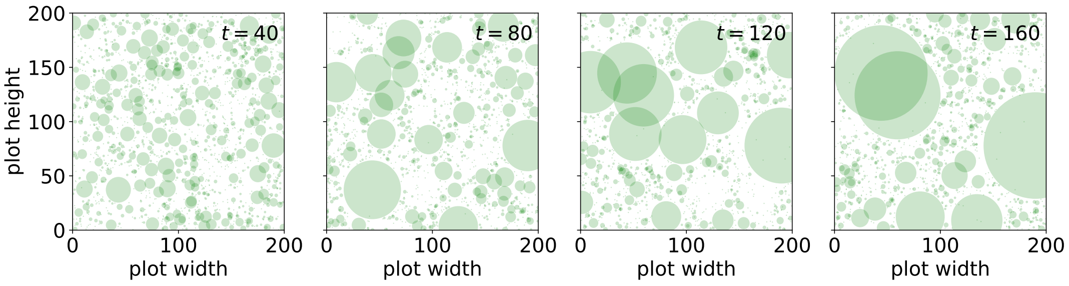

Fig. S7. Change in plots over time corresponding to those shown in Figure S6 when starting from an initially empty plot. Green circles show canopy extent.

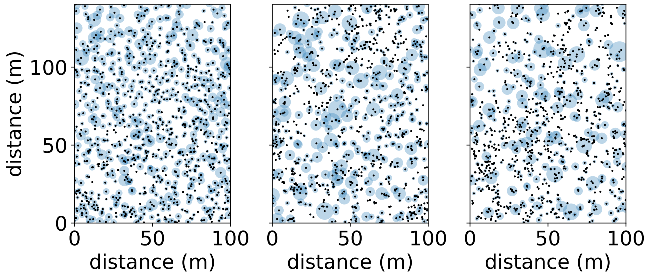

Fig. S8. Examples of variation in spatial location in Alaskan rainforest plotted using data from reference (15). Radius of blue circles reflect reported basal stem radius though on an exaggerated scale for visibility.

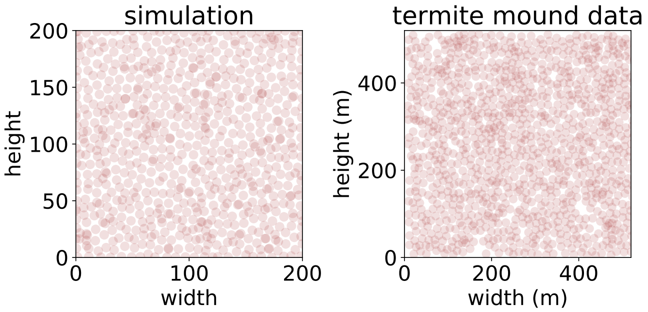

Fig. S9. Packed, "solid" regime in simulation compared with termite-mound packing in Namibia from reference (16). See Figure 4D for corresponding spatial correlation functions. As revealed by the neighbor density function there, termite-mound packing is tighter than what our simulation recovers by about 10% given the observed density (circles indicating termite mounds on right graph are meant to be demonstrative and not indicative of actual size or foraging range).

the steady state with a small correction $\epsilon u(r)$ to obtain

$$\begin{aligned}\epsilon\dot{u}(r,t) = -\partial_r\left(\dot{r}(r)[\bar{n}(r)+\epsilon u(r,t)]\right) - \mu(r)[\bar{n}(r)+\epsilon u(r,t)] - \\ \epsilon\bar{n}(r)a(r)\int_{r_0}^{r_{\max}} u(r',t)a(r')\,dr' - \\ \epsilon u(r,t)a(r)\int_{r_0}^{r_{\max}} \bar{n}(r')a(r')\,dr',\end{aligned} \qquad \text{[S23]}$$

having only kept terms up to linear order of the perturbation $\epsilon\, u(r,t)$ since $\epsilon \ll 1$. Though we consider the specific example of symmetric competition in Eq S23, a similar derivation applies to the asymmetric case.

By equating the terms linear in ϵ, we obtain the following equation for perturbations at each radius r,

$$\begin{aligned}\dot{u}(r,t) = -\partial_r[\dot{r}(r)u(r,t)] - \mu(r)u(r,t) - \\ \bar{n}(r)a(r)\int u(r',t)a(r')\,dr' - u(r,t)a(r)\int \bar{n}(r')a(r')\,dr'.\end{aligned} \qquad \text{[S24]}$$

Eq S24, because it is linear in time, admits general solutions of the form $u(r,t) = A(r)e^{\lambda(r)t}$, where $\lambda(r)$ determines how perturbations about the steady steady solution behave. This can be determined from the characteristic polynomial of Eq S24. The real part of $\lambda(r)$ determines if perturbations grow, $\Re[\lambda(r)] > 0$, or if they decay, $\Re[\lambda(r)] < 0$. When the imaginary component is nonzero, then perturbations will generate oscillations.

From numerical calculation of the eigenvalues $\lambda(r)$, we find that typically $\lambda(r)$ is complex when there are competitive interactions, whether interactions are symmetric or asymmetric. Furthermore, we often find that the oscillations are heavily damped though there are some regimes of parameter space showing long persistent oscillations like in Figure 5. Interestingly, we also find that there are regimes in phase space where $\Re[\lambda(r)] > 0$, indicating unstable directions that could lead to an alternative steady states. Though this mean-field approximation does not completely capture the stochastic 2D simulation, we find similar oscillations as shown in Figure S6, indicating that population waves are a generic feature arising from delayed self-coupling of populations from metabolic growth.

I. Survey plot data sets

In Figure S8, we show additional forest plots from the references cited in the main text for Alaskan rainforests. Though these all constitute similar ecosystems, there is variation between location that may depend on factors like local geography. These effects may be expressed in our parameters for competitive strength and resource fluctuations.

In Figure S9, we compare the hexagonal packing found in termite mounds with our simulation.

1. BJ Enquist, GB West, JH Brown, Extensions and evaluations of a general quantitative theory of forest structure and dynamics. *Proc. Natl. Acad. Sci. U.S.A.* **106**, 7046–7051 (2009).
2. CP Kempes, GB West, K Crowell, M Girvan, Predicting Maximum Tree Heights and Other Traits from Allometric Scaling and Resource Limitations. *PLoS ONE* **6**, e20551 (2011).
3. A Mrad, et al., Recovering the Metabolic, Self-Thinning, and Constant Final Yield Rules in Mono-Specific Stands. *Front. For. Glob. Chang.* **3**, 62 (2020).
4. KJ Niklas, BJ Enquist, Invariant scaling relationships for interspecific plant biomass production rates and body size. *Proc. Natl. Acad. Sci. U.S.A.* **98**, 2922–2927 (2001).
5. S Mori, et al., Mixed-power scaling of whole-plant respiration from seedlings to giant trees. *Proc. Natl. Acad. Sci.* **107**, 1447–1451 (2010).
6. O Peters, JD Neelin, Critical phenomena in atmospheric precipitation. *Nat. Phys* **2**, 393–396 (2006).
7. NCDC, Climate Data Online (https://www.ncdc.noaa.gov/cdo-web/) (2020).

8. GB West, BJ Enquist, JH Brown, A general quantitative theory of forest structure and dynamics. *Proc. Natl. Acad. Sci. U.S.A.* **106**, 7040–7045 (2009).
9. Si Amari, *Information Geometry and Its Applications*, Applied Mathematical Sciences. (Springer Japan) Vol. 194, (2016).
10. WS Bialek, *Biophysics: Searching for Principles*. (Princeton University Press, Princeton, NJ), (2012).
11. TM Cover, JA Thomas, *Elements of Information Theory*. (John Wiley & Sons, Hoboken), Second edition, (2006).
12. HC Muller-Landau, et al., Testing metabolic ecology theory for allometric scaling of tree size, growth and mortality in tropical forests. *Ecol. Lett.* **9**, 575–588 (2006).
13. T Erneux, *Applied Delay Differential Equations*. (Springer New York, New York, NY) Vol. 3, (2009).
14. SM Sah, RH Rand, Three Ways of Treating a Linear Delay Differential Equation in *Recent Trends in Applied Nonlinear Mechanics and Physics*, ed. M Belhaq. (Springer International Publishing, Cham) Vol. 199, pp. 251–257 (2018).
15. EE Schneider, JS Crotteau, AJ Larson, Southeast Alaska old-growth forest stem map data collected in 1964 on ten 1.42 hectare plots (2020).
16. CE Tarnita, et al., A theoretical foundation for multi-scale regular vegetation patterns. *Nature* **541**, 398–401 (2017).
